# Therapeutic potential of plant-derived natural products against drug-induced liver injury

**DOI:** 10.3389/fphar.2025.1652860

**Published:** 2025-09-01

**Authors:** Yan Chen, Yu-Qi Mei, Lin Hou, Ke-Jian Li

**Affiliations:** ^1^ Research Institute of Marine Traditional Chinese Medicine, Shandong University of Traditional Chinese Medicine, Qingdao, China; ^2^ Qingdao Academy of Chinese Medical Sciences, Shandong University of Traditional Chinese Medicine, Qingdao, China; ^3^ Institute of Chinese Materia Medica, Shanghai University of Traditional Chinese Medicine, Shanghai, China; ^4^ College of Traditional Chinese Medicine, Shandong University of Traditional Chinese Medicine, Jinan, China

**Keywords:** flavonoids, phenylpropanoids, terpenoids, glycosides, drug-induced liver injury, mechanism

## Abstract

Drug-induced liver injury (DILI) is a major cause of drug development failure and post-marketing restrictions. To date, over 1,000 drugs have been reported to cause liver damage, such as acetaminophen, isoniazid, methotrexate, triptolide and so on. However, there are currently no effective therapies for DILI. Plant-derived natural products including flavonoids, phenylpropanoids, terpenoids, and glycosides have been used for the treatment of DILI due to their low toxicity and strong bioactivity. These anti-DILI compounds involve multiple mechanisms, such as reducing oxidative stress and inflammation, restoring mitochondrial function, and suppressing apoptosis. This review primarily summarizes the recent advances over the past 5 years in the therapeutic potential of natural products against a range of commonly used hepatotoxic drugs rather than focusing on a specific hepatotoxic agent. The insights will provide a cue for further research and promote the development of novel and effective drugs for treating DILI.

## Graphical Abstract

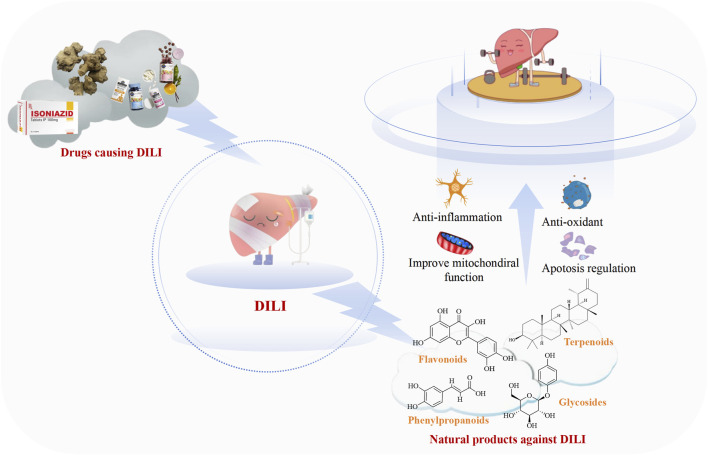

## 1 Introduction

**FIGURE 1:**
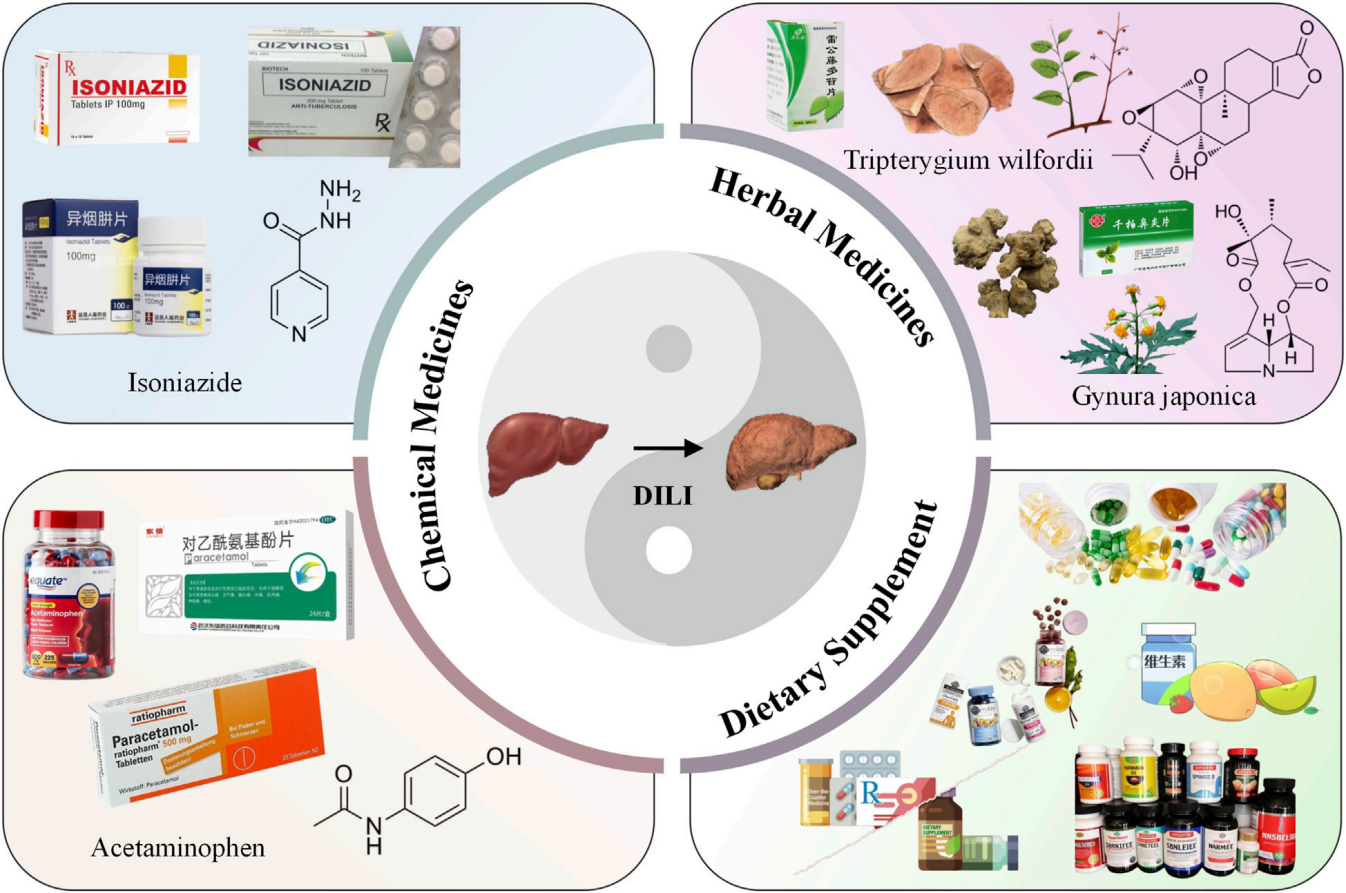
Liver injury caused by commonly used medications.

The liver accounts for approximately 2% of the human body weight, and it performs many vital functions including metabolism, synthesis, detoxification, immunity, hematopoiesis, blood storage, blood volume regulation, and coagulation (Berasain et al., 2023; Li J. et al., 2024). However, many commonly used medications pose a high risk of causing liver injury, which is an uncommon but challenging clinical problem with respect to both diagnosis and treatment (Hoofnagle and Björnsson, 2019). Drug-induced liver injury (DILI) remains a leading cause of drug development termination and post-marketing warnings and restriction of use. To date, over 1,000 drugs have been reported to cause liver injury, which can develop into necrosis, cirrhosis, liver failure and cancer, or even death (Ma et al., 2023; Neshat et al., 2021). Both prescription and non-prescribed drugs may harm the liver, including medications, herbal medicines, and dietary supplements (Garcia-Cortes et al., 2020; Real et al., 2019; Figure 1). According to the statistics, the incidence of DILI is 14–19/100,000 people in Western countries, and it is the most common cause of acute liver failure (Hosack et al., 2023; Björnsson and Björnsson, 2022; European Association for the Study of the Liver, 2019). Meanwhile, in China, it is estimated that the annual occurrence in the general population is approximately 23.80/100,000, which is higher than that reported in Western countries (Ma et al., 2023). It has a low incidence among the general population, but DILI has become more prevalent in hospitalized patients, especially among patients with unexplained liver conditions (Hosack et al., 2023; Li et al., 2022). In addition, smoking, alcohol consumption, viral infections, and drug–drug interactions can exacerbate DILI (Rani et al., 2024). Although the past decade has witnessed major efforts in the prevention and treatment of other liver diseases, progress on these fronts has been modest in the case of DILI (Devarbhavi et al., 2023).

Natural products derived from diverse sources, including plants, animals, microorganisms, fermentation products, and marine organisms, exhibit a broad spectrum of biological activities and chemical structures. These compounds hold significant potential as alternative or adjunctive therapeutic agents (Rao et al., 2019; Atanasov et al., 2021). The medicinal application of natural products can be tracked to the origins of traditional Chinese medicine. In recent years, with the increasing scientific interest in natural products among researchers, an increasing number of natural products have been identified as having beneficial effects on liver diseases (Aboelez et al., 2024; Abouzed et al., 2024). Plant-derived flavonoids, terpenoids, phenylpropanoids, and glycosides have validated hepatoprotective properties (Sahu et al., 2023; Xu G. B. et al., 2018; Guo C. et al., 2024; Thilagavathi et al., 2023). For instance, silymarin, a phenylpropanoid from *Silybum marianum* (L.) Gaertn., has been utilized in alcoholic or nonalcoholic fatty liver disease and drug-induced liver injury (Gillessen and Schmidt, 2020). Similarly, schizandrin A and B from *Schisandra chinensis* (Turcz.) Baill., have been shown to exert hepatoprotective effects, particularly in the prevention and treatment of liver diseases (Zhang X. et al., 2020). However, despite the fact that natural products and their derivatives accounted for over one-third of all FDA-approved new molecular entities in the past 5 years (Luo et al., 2024), few natural products have been approved specifically for combating DILI by FDA. In this review, we comprehensively summarized the recent advances in the field of hepatoprotective effects of natural products, aiming to provide valuable insights for future research and facilitate the development of novel, effective therapeutics against DILI.

## 2 The classification and pathogenesis characteristics of DILI

Based on the pathogenesis, DILI is typically classified into two types: intrinsic (direct) and idiosyncratic. However, indirect injury has been increasingly recognized as a third type. The characteristics of DILI pathogenesis are shown in Table 1 (Hoofnagle and Björnsson, 2019; Ma et al., 2023). Intrinsic liver injury is caused by medications or substances that are intrinsically toxic to the liver. Common causative agents include acetaminophen, nicotinic acid, aspirin, cocaine, amiodarone, methotrexate, certain chemotherapeutic drugs, and traditional Chinese medicines containing pyrrolizidine alkaloids. This type of liver injury is common, predictable, and dose-dependent. It typically has a short latency period and presents clinically as acute hepatitis (Garcia-Cortes et al., 2020; Maris et al., 2025). In contrast, idiosyncratic DILI is often described as being unpredictable and not dose-related (Ma et al., 2023). Although idiosyncratic liver injury is not considered dose-dependent, such injury is more commonly associated with orally administered drugs at daily doses ≥50 mg or with agents capable of triggering immune-mediated reactions (Björnsson and Björnsson, 2022). Unlike the above two types of liver injury, indirect drug-induced liver injury arises from the effects of a drug rather than from intrinsic hepatotoxicity or an idiosyncratic reaction to the medication. For example, protein kinase inhibitors and monoclonal antibodies can induce immune-mediated liver injury. Additionally, indirect injury may manifest as the onset of a new liver condition or exacerbation of a pre-existing condition (Hoofnagle and Björnsson, 2019).

**TABLE 1:** Characteristics of DILI pathogenesis (Ma et al., 2023).

Characteristic	Intrinsic (direct)	Idiosyncratic	Indirect
Dose-related	Yes	No	No
Predictable	Yes	No	Partially
Latency	Short (few days)	Variable (days to months)	Delayed (months)
Mechanism	Drugs or metabolites cause intrinsic hepatotoxicity	Metabolic damage or immune-mediated hepatotoxicity	Liver injury caused by modification of the underlying disease or immune state
Typical phenotypes	Acute hepatitis	Acute, mixed, or cholestatic hepatitis	Acute hepatitis, autoimmune hepatitis, or fatty liver
Common drugs	Acetaminophen, nicotinic acid, aspirin, cocaine, amiodarone, methotrexate, cancer chemotherapy, and plants containing pyrrolizidine alkaloids	Isoniazid, macrodantin, amoxicillin-clavulanate, minocycline, ketoconazole, fenofibrate, fluoroquinolones, and macrolides	Antineoplastic drugs, glucocorticoids, protein kinase inhibitors, and monoclonal antibodies

## 3 Plant-derived natural anti-DILI products

### 3.1 Flavonoids

Flavonoids are natural polyphenolic compounds, characterized by a general structure consisting of two benzene rings (Shen et al., 2022). At present, more than 5,000 flavonoids have been identified in different sources, mainly from the Compositae, Lamiaceae, Rutaceae, and Scrophulariaceae families, which show extensive bioactivities (Wen et al., 2021; Li C. H. et al., 2023). Both *in vivo* and *in vitro* studies exhibited the potential effect of flavonoids in preventing and treating liver diseases.

#### 3.1.1 Quercetin

Quercetin, a well-known natural antioxidant, is widely present in vegetables, fruits, and medicinal plants (Andres et al., 2018). Rats were given quercetin (25 mg/kg or 50 mg/kg p.o.) for 14 days, which decreased vincristine-induced liver injury via modulating the levels of nuclear factor erythroid 2-related factor 2 (Nrf2)/hemeoxygenase-1 (HO-1), SIRT1/PGC-1α, and NF-kB/STAT3 (Çomaklı et al., 2023). These results suggested that the liver-protective activities are closely related to its potent antioxidant properties and multi-pathway regulatory capacity.

#### 3.1.2 Baicalin

Baicalin is the main compound from the Chinese medicinal plant *Scutellaria baicalensis* Georgi, which possess various pharmacological activities such anti-inflammatory, antibacterial, antiviral, and antioxidant activities (Hou et al., 2024). Furthermore, several studies showed that baicalin plays an important therapeutic role in liver disease, including DILI (Hu M. L. et al., 2021; Yang et al., 2021). Baicalin promotes hepatocyte proliferation after acetaminophen (APAP)-induced liver injury (Shi et al., 2020; Zhang et al., 2024). It can induce the accumulation of Nrf2 (Shi et al., 2018; 2020), subsequently activating the NOD-like receptor pyrin domain-containing 3 (NLRP3) inflammasome (Shi et al., 2020). Furthermore, baicalin mediates the mammalian target of rapamycin (mTOR) signaling pathway, thereby promoting liver repair in APAP-induced liver injury (Zhang et al., 2024). Taken together, these findings suggest that liver regeneration is likely the primary mechanism by which baicalin exerts its protective effects against DILI.

#### 3.1.3 Kaempferol

Kaempferol, a flavonoid predominantly isolated from the rhizome of *Kaempferia galanga* L., is also ubiquitously distributed in various vegetables and fruits. It has been extensively studied for its various biological activities such as anti-oxidation, cancer prevention, neuroprotection, and hepatoprotection (Amjad et al., 2022; Dong et al., 2023; Bangar et al., 2023). Rats were orally administered 250 mg/kg kaempferol for 7 days, which activated the silent information regulator 1 and decreased the acetylation of peroxisome proliferators-activated receptor-γ (PPAR-γ), forkhead transcription factors-1 (FOXO-1), nuclear factor-κB (NF-κB), and p53 in the APAP-induced liver injury model (BinMowyna and AlFaris, 2021). Additionally, Li et al. showed that kaempferol mediated the Nrf2 pathway and upregulated the levels of glutathione peroxidase 4 (Gpx4) in mouse liver and L02 cells to inhibit ferroptosis induced by APAP (Li Y. G. et al., 2023). Similar to other flavonoids, the role of kaempferol in DILI is related to its antioxidant capacity, which may be attributed to the presence of phenolic hydroxyl groups in its structure.

#### 3.1.4 Hyperoside

As a quercetin-derived flavonol galactoside, hyperoside, also known as quercetin-3-O-galactoside, is extensively found in Hypericaceae, Rosaceae, Campanulaceae, and Lamiaceae members. It exhibits multiple pharmacological properties, including antioxidant, anti-inflammatory, anti-cancer, cardioprotective, and neuroprotective effects (Xu et al., 2022). Notably, hyperoside attenuated liver damage caused by APAP, pyrrolizidine alkaloids (PAs), and the chemotherapeutic agent cisplatin (Xie et al., 2016a; Hu C. et al., 2020; Xu et al., 2023a; Niu et al., 2017). Xie and Hu et al.’s studies suggested that hyperoside protects APAP-induced liver injury by regulating the glutathione pathway and suppressing the activity of Cyp2e1 in mice (Xie et al., 2016a; Hu F. F. et al., 2020). Furthermore, it has also been shown to exert a protective effect against PA-induced liver injury by ameliorating transcription factor EB-mediated mitochondrial dysfunction (Xu et al., 2023a). Although hyperoside was beneficial for various DILI models, its *in vitro* and *in vivo* mechanism still needs to be further explored.

#### 3.1.5 Naringin

Naringin is a dihydroflavonoid that is mainly found in the Rue family, such as grapefruits and oranges. It is also a main component of traditional Chinese medicine, including Drynariae Rhizoma, Aurantii Fructus, Aurantii Fructus Immaturus, and Citri Grandis Exocarpium (Yang et al., 2022). Naringin has been reported to exert various biological and pharmacological effects (Chen J. et al., 2024; Zhu et al., 2024; Heidary et al., 2020; Ahmed et al., 2019). Growing evidence suggests that naringin exerts antioxidant and anti-inflammatory effects that are effective in the treatment of liver diseases (Zhu et al., 2024; Shirani et al., 2020). Recent studies have shown that naringin can also ameliorate multi drug-induced hepatotoxicity, particularly against chemotherapeutic agents. A prominent example is doxorubicin, a highly effective anticancer drug whose clinical application is significantly constrained by severe dose-dependent organ toxicity, including hepatotoxicity, cardiotoxicity, nephrotoxicity, and neurotoxicity (Pugazhendhi et al., 2018). Xi et al. found that naringin attenuates doxorubicin-induced liver injury by upregulating the expression levels of sirtuin (SIRT1) and inhibiting the downstream inflammatory, apoptotic, and oxidative stress signaling pathways in mice and in alpha mouse liver 12 (AML-12) cells (Xi et al., 2023). In addition, naringin alleviates gefitinib-, methotrexate-, and oxaliplatin-induced liver injury through anti-oxidation and inhibition of autophagy and apoptosis (Liu et al., 2024; Elsawy et al., 2020; Ileriturk et al., 2024); however, the specific molecular mechanisms and signaling pathways require further investigation. Moreover, naringin was found to upregulate the expression of cation transport regulator-like protein 2 (CHAC2) and activate the Nrf2 pathway, thereby exerting a protective effect against APAP-induced liver injury (Zhai et al., 2022). Collectively, these findings highlight the potential of naringin as a natural therapeutic agent for the prevention or treatment of DILI.

#### 3.1.6 Other flavonoids

**FIGURE 2:**
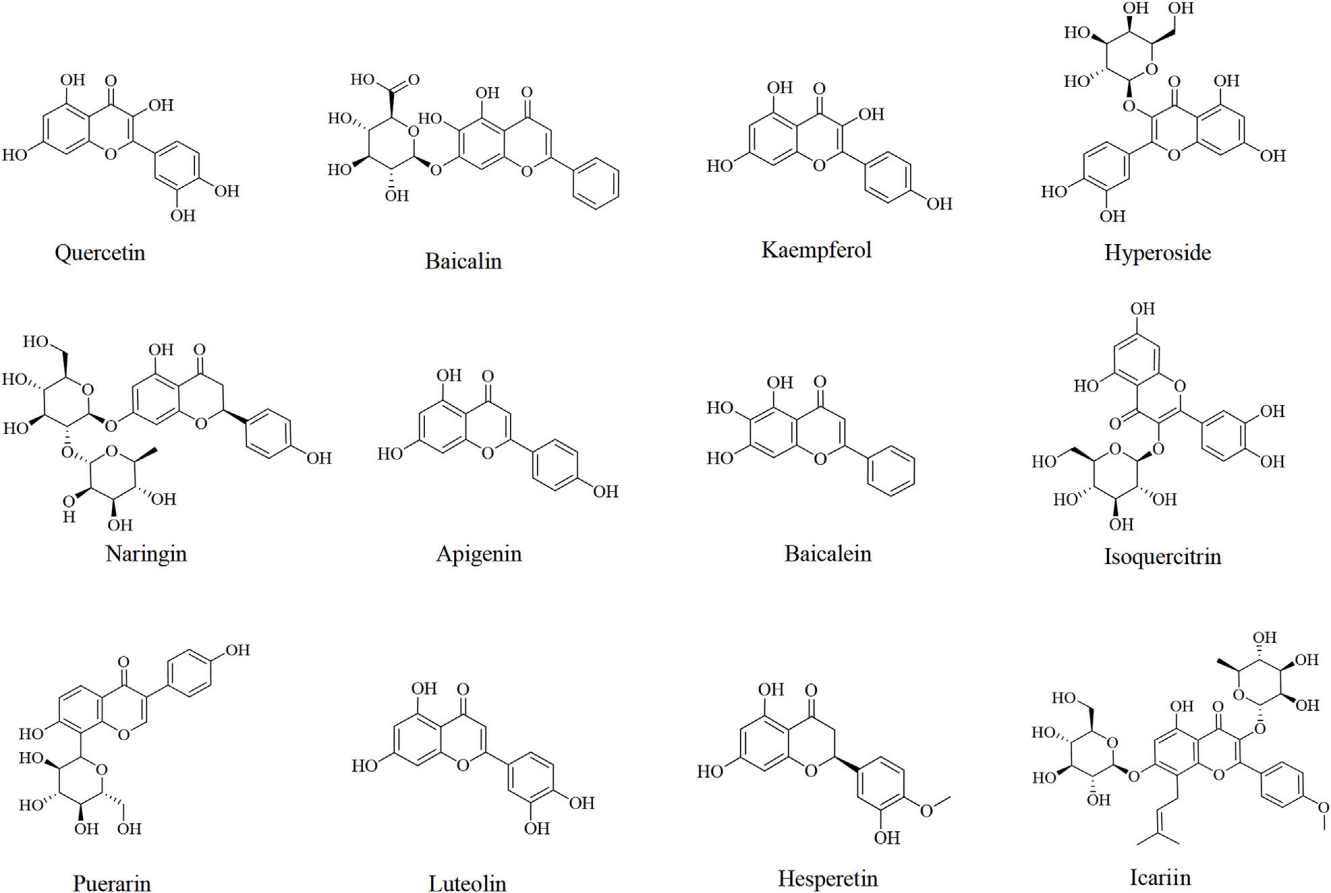
Chemical structures of flavonoids against DILI.

Other flavonoids including apigenin (Meng et al., 2022; Sahindokuyucu-Kocasari et al., 2021; Zhang W. et al., 2020), baicalein (Shi et al., 2017), isoquercitrin (Xie et al., 2016b), puerarin (Zhou et al., 2023), luteolin (Tai et al., 2015), hesperetin (Wang et al., 2020), and icariin (Shen et al., 2024) have also been studied for their potential in the prevention and treatment of DILI, which are listed in Table 2 and Figure 2.

**TABLE 2:** Natural products for the prevention and treatment of drug-induced liver injury.

Structure	Chemical compound	Models	Related targets/pathways	Reference
Flavonoids	Quercetin	Vincristine (rats)	Nrf2, HO-1, SIRT1, PGC-1α, NF-kB, and STAT3	Çomaklı et al. (2023)
	Baicalin	APAP (mice)	Nrf2, NLRP3, and mTOR	Shi et al. (2018), Shi et al. (2020) Zhang et al. (2024)
	Kaempferol	APAP (rat)	SIRT1, PPAR-γ, FOXO-1, NF-κB, p53, and Gpx4	BinMowyna and AlFaris (2021) Li Y. G. et al. (2023)
	Hyperoside	APAP (mice) PAs (mice)	Cyp2e1	Xie et al. (2016a) Hu et al. (2020a) Xu et al. (2023a)
	Naringin	Cyclophosphamide (rats) Gefitinib (mice) Methotrexate (rats) Oxaliplatin (rats)	SIRT1,CHAC2, Bcl-2, Bax, and TGF-β1	Xi et al. (2023) Liu et al. (2024) Elsawy et al. (2020) Ileriturk et al. (2024) Zhai et al. (2022)
	Apigenin	Methotrexate (mice) APAP (mice)	AMPK	Meng et al. (2022) Sahindokuyucu-Kocasari et al. (2021) Zhang X. et al. (2020)
	Baicalein	APAP (mice) Monocrotaline (rats)	ERK1/2, Nrf2, protein kinase C (PKC), PI3K, and MAPK	Shi et al. (2018) Shi et al. (2017)
	Isoquercitrin	APAP (mice)	CYP2E1, SULT, NF-κB, and MAPK	Xie et al. (2016b)
	Puerarin	APAP (mice)	Nrf2 and Keap1	Zhou et al. (2023)
	Luteolin	APAP (mice)	NF-kB	Tai et al. (2015)
	Hesperetin	APAP (mice)	HO-1 and TLR-4	Wang et al. (2020)
	Icariin	APAP (mice)	S100A9	Shen et al. (2024)
Phenylpropanoids	Chlorogenic acid	APAP (mice) Methotrexate (rats)	Nrf2, HSP60, EGR1, Bax, and Bcl-2	Xue et al. (2023) Hu et al. (2020b) Wei et al. (2023a) Owumi et al. (2021)
	Ferulic acid	Tamoxifen (rats) APAP (mice) Cyclosporine (rats) Diosbulbin B (mice)	AMPK, Nrf2/HO-1, and IL-1β	Li et al. (2021) Wu et al. (2022) Nouri et al. (2023)
	Schisandrin B	APAP (rats) Clozapine (mice)	EGFR, TFEB, MAPK, JUK, ERK, Nrf2, and ARE	Nasser et al. (2020) Li H. Y. et al. (2023) Cheng et al. (2022)
	Schisandrin A	Senecionine (mice)	CYP3A4	Bai and Feng (2017)
	Caffeic acid	APAP (mice)	ERK1/2, Keap1, and Nrf2	Chen et al. (2022) Pang et al. (2016a)
	Salvianolic acid B	Senecionine (mice) APAP (mice)	TGF-β1, STAT3, Nrf2, PI3K, and PKC	Pang et al. (2016b) Ye et al. (2021)
Terpenoids	Andrographolide	APAP (mice) Monocrotaline (rats)	Nrf2 and HO-1	Qin et al. (2023) Yan et al. (2018)
	Taraxasterol	APAP (mice)	Nrf2	Xu L. et al. (2018) Lin et al. (2024)
	Glycyrrhetinic acid	Retrorsine (mice) Diosbulbin B (mice) APAP (mice)	Nrf2, PI3K, Akt, GSK3β, CYP3A4, CYP2E1, HMGB1, and TLR4	Teschke (2014) Wang et al. (2022) Lin et al. (2023)
	Ursolic acid	Tetrandrine	GST	Zhou H. M. et al. (2024)
	Catalpol	Triptolide (HepaRG cell/mice)	PERK, ATF4, CHOP, SIRT1, and HIF-1α	Chu et al. (2022) Nie et al. (2024)
	Geniposidic acid	APAP/*Tripterygium wilfordii*(mice/AML12 cell/L02 cell)	FXR	Fan et al. (2025)
	Geniposide	Tripterygium glycosides (mice)	GST	Zhang et al. (2022)
	Alisol B 23-acetate	Senecionine (mice)	AQP2	Wang et al. (2016)
	Oleanolic acid	APAP (mice)	MAPK	Tang et al. (2024) Yong et al. (2019)
Glycosides	Arbutin	Cyclophosphamide (rats)	Nrf2/HO-1	Okkay et al. (2024)
	Salidroside	APAP (mice)	Sirt1, Akt, Nrf2, NF-κB, and NLRP3	Wu et al. (2008) Gao et al. (2023)
	Gastrodin	APAP (mice)	MAPK and Nrf2	Xiao et al. (2023)
	Ginsenoside Rg1	APAP (mice)	Nrf2	Guo et al. (2024c)
	Ginsenoside Rh1	APAP (mice)	Nrf2	Ning et al. (2018)
	Ginsenoside Rb1	Cantharidin (mice)	Caspase-3/8, Bcl-2, Bax, GRP78, ATF6, ATF4, CHOP, IRE1α, and PERK	Liu et al. (2020)
	Echinacoside	APAP (mice)	CYP2E1	Gao et al. (2017)
	Paeoniflorin	APAP (mice)	MAPK, mTOR, and JNK	Thida et al. (2021) Deng et al. (2024)
	Notoginsenosides	APAP (mice)	TNFα	Deng et al. (2022)
	Escin	APAP (mice)	ERK	Tian et al. (2024)
	Crocin	Leflunomide (mice)	TLR4, PI3K, and mTOR	Sokar et al. (2022)
	Astragaloside IV	Cisplatin (mice)	PPARα	Guo et al. (2024a)

### 3.2 Phenylpropanoids

Phenylpropanoids, which include phenylpropionic acid, phenylpropenes, phenylpropanols, phenylpropionaldehydes, coumarins, lignans, and lignins, are characterized by a core C_6_–C_3_ carbon skeleton (Li et al., 2025). As a major class of phenolic compounds, phenylpropanoids are widely distributed in plants and exhibit a broad range of biological activities such as antioxidant, anti-inflammation, anti-cancer, neuroprotective, cardioprotective, and hepatoprotective effects (Neelam et al., 2020).

#### 3.2.1 Chlorogenic acid

Chlorogenic acid, a typical phenolic acid, is formed by a conjugation between the hydroxy group of quinic acid and the carboxyl group of caffeic acid. It is commonly derived from the Caprifoliaceae family plants such as *Lonicera japonica* Thunb. (Nguyen et al., 2024). Previous studies have demonstrated that chlorogenic acid exerts protective effects against DILI both *in vitro* and *in vivo* (Pang et al., 2015; Zheng et al., 2016; Xue et al., 2023). Nrf2 plays a key role in the hepatoprotective effects of chlorogenic acid, which alleviates APAP-induced liver injury by regulating heat shock protein 60 (HSP60)-initiated liver inflammation (Hu F. F. et al., 2020). Another study showed that chlorogenic acid promotes liver regeneration and repair in APAP-intoxicated mice by transcriptionally activating the early growth response-1 (EGR1) (Wei M. J. et al., 2023). Furthermore, chlorogenic acid has been reported to alleviate tamoxifen- and methotrexate-induced liver injury in rats by mitigating inflammation and apoptosis and enhancing the antioxidant defense (Owumi et al., 2021; Ali et al., 2017). However, the underlying mechanisms of these effects have not yet been fully elucidated.

#### 3.2.2 Ferulic acid

Ferulic acid is widely found in plants of the Umbelliferae, Ranunculaceae, and Liliaceae families, such as *Ligusticum chuanxiong* Hort., *Angelica sinensis* (Oliv.) Diels., and *Cimicifuga heracleifolia* Kom. (Zhang and Gao, 2020). It possesses multiple bioactivities, with particularly notable antioxidant and anti-inflammatory properties (Li et al., 2021). Ferulic acid holds great potential in alleviating DILI due to its characteristics. Treatment with 25 mg/kg ferulic acid three times per 12 h is shown to restore liver function to normal levels in mice with APAP-induced liver injury, along with the upregulation of hepatic specific markers and AMPK phosphorylation. Additionally, ferulic acid ameliorated APAP-induced mitochondrial damage and apoptosis in hepatocytes (Wu et al., 2022). It also activated the Nrf2/hemeoxygenase-1 (HO-1) signaling axis and decreased the expressions of inflammatory cytokines including NF-kB, TNFα, and interleukin-1β (IL-1β) (Nouri et al., 2023). These findings demonstrated that the hepatoprotective effects of ferulic acid are closely related to its antioxidant capacity. Moreover, Chen et al. reported that ferulic acid mitigated diosbulbin B-induced hepatotoxicity by reducing the formation of reactive metabolite protein adducts (Chen et al., 2023).

#### 3.2.3 Schisandrin B

Schisandrin B is one of the main lignan compounds isolated from the traditional Chinese medicine *Schisandra chinensis* (Turcz.) Baill. Numerous studies have shown that schisandrin B plays an essential role in liver protection (Nasser et al., 2020). Li et al. found that schisandrin B inhibited the production of TNF-α and IL-1β; upregulated the expression levels of beclin-1, transcription factor EB (TFEB), and LC-3; and downregulated the expressions of autophagy-related protein 3 (ATG3) and epidermal growth factor receptor (EGFR) in APAP-treated HepG2 cells (Li H. Y. et al., 2023). It has also been reported that schisandrin B can not only activate the pentose phosphate pathway but also suppress the mitogen-activated protein kinase (MAPK)-c-Jun N-terminal kinase (JNK)-extracellular signal-regulated kinase (ERK) signaling pathway in an APAP-induced liver cell line (HHL-5 cells) (Cheng et al., 2022). In addition, schisandrin B was found to have protective effects against clozapine-induced liver injury via the activation of the Nrf2/antioxidant response element (ARE) signal pathway (Bai and Feng, 2017). Although these studies suggested that schisandrin B can alleviate DILI, its *in vivo* mechanism of action remains unclear.

#### 3.2.4 Other phenylpropanoids

**FIGURE 3:**
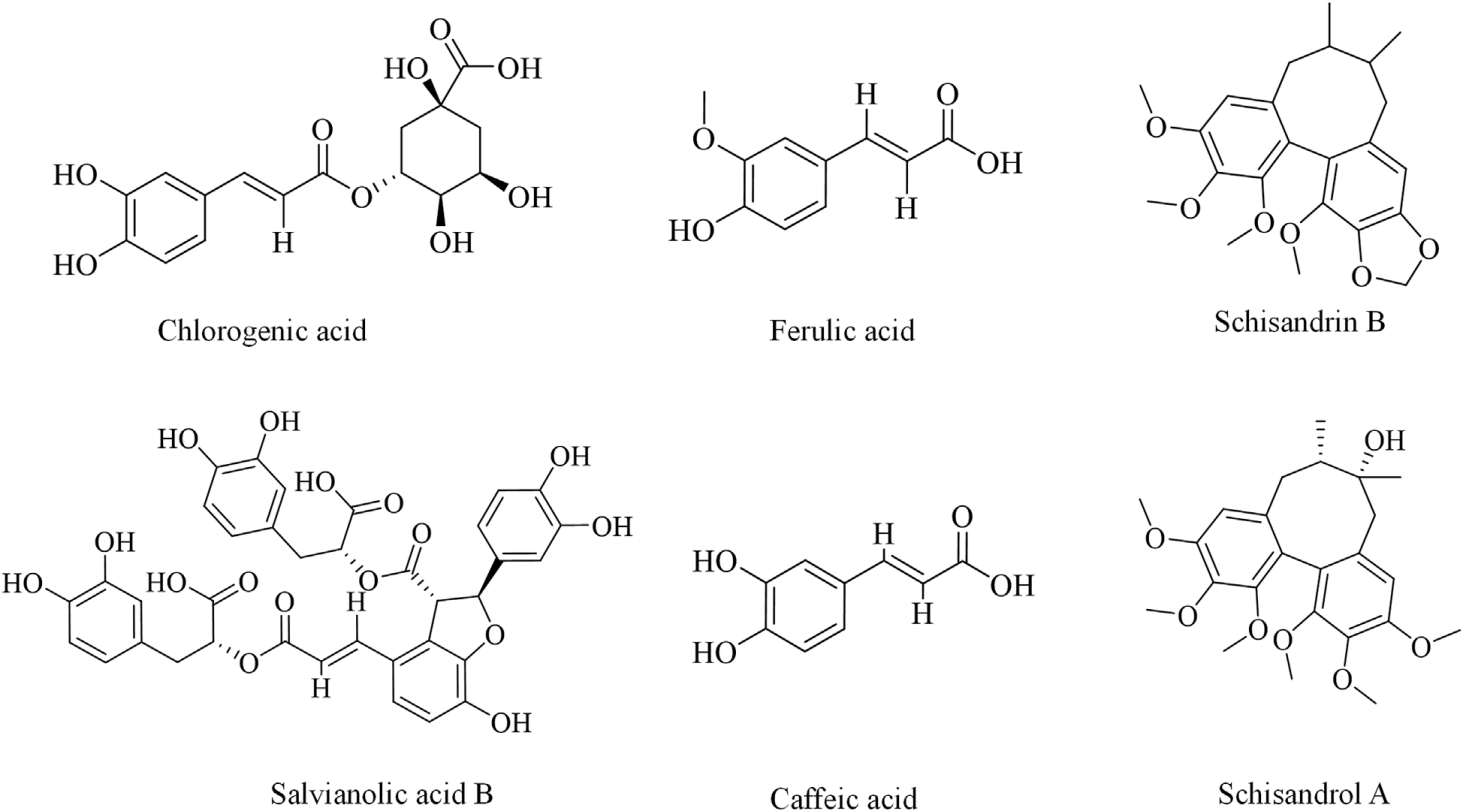
Chemical structures of phenylpropanoids against DILI.

Other phenylpropanoids including schisandrin A (Chen et al., 2022), caffeic acid (Pang et al., 2016a; Pang et al., 2016b), and salvianolic acid B (Ye et al., 2021; Lin et al., 2015) have also been reported to possess significant potential in the prevention and treatment of DILI, as summarized in Table 2 and illustrated in Figure 3.

### 3.3 Terpenoids

Terpenoids are a class of active natural products characterized by a complex structure and diverse biological activities. Based on the number of isoprene units in their chemical structure, terpenoids are classified into several subclasses, including hemiterpenoids, monoterpenoids, sesquiterpenoids, diterpenoids, sesterterpenes, triterpenoids, tetraterpenes, and polyterpenes (Kuang, 2017). The hepatoprotective potential of terpenoids in the prevention and treatment of liver diseases has attracted considerable attention (Wei J. R. et al., 2023; Yao and Liu, 2022).

#### 3.3.1 Andrographolide

Andrographolide is a diterpenoid compound isolated from *Andrographis paniculata* (Burm.f.) Nees, and it has been clinically used for the treatment of upper respiratory tract infection (Hossain et al., 2021; Zeng et al., 2022). Recent studies have showed that andrographolide exerts a curative effect in liver diseases (Qin et al., 2023). Long-term ingestion of APAP can induce liver fibrosis, while andrographolide has been shown to alleviate APAP-induced liver fibrosis in mice by activating Nrf2 and upregulating the expression of downstream genes glutamate-cysteine ligase (GCLC and GCLM) and heme oxygenase-1 (HO-1) (Yan et al., 2018). These results suggested that the hepatoprotective effect of andrographolide was closely associated with its antioxidant capacity. Additionally, Huang et al. indicated that andrographolide can attenuate monocrotaline-induced hepatotoxicity by modulating Nrf2-dependent mitochondrial biogenesis and antioxidant responses (Huang et al., 2023).

#### 3.3.2 Taraxasterol

Taraxasterol is a pentacyclic triterpenoid compound with strong antioxidant and anti-inflammatory activities derived from *Taraxacum mongolicum* (Jiao et al., 2022). Previous studies have indicated that taraxasterol exhibits protective effects in various liver diseases (Sang et al., 2019; Xu L. et al., 2018; Lin et al., 2024; Ge et al., 2023). In APAP-treated mice and cell models, taraxasterol was shown to restore the expression of Nrf2 and alleviate APAP-induced cellular injury. In addition, Lin et al. further revealed that taraxasterol decreases CYP1A1 expression and increases UGT1A1 expression (Lin et al., 2024). In another study, Ge et al. reported that taraxasterol markedly suppresses APAP-induced liver oxidative stress, inflammatory responses, and apoptosis. The underlying mechanisms were related to the modulation of Nrf2/HO-1 and JNK phosphorylation (Ge et al., 2023).

#### 3.3.3 Glycyrrhetinic acid

Glycyrrhetinic acid, a hydrolyzed metabolite of glycyrrhizic acid *in vivo,* is one of the prominent active compounds of *Glycyrrhiza uralensis* (Pastorino et al., 2018). It is also a pentacyclic triterpenoid with hepatoprotective, antioxidant, and anti-tumor effects, which have attracted considerable attention among scientists (Chen Y. et al., 2024). Owing to the well-documented liver-protective properties of *Glycyrrhiza uralensis*, numerous studies have suggested that glycyrrhetinic acid may contribute significantly to its hepatoprotective potential (Wu et al., 2021; Jiang et al., 2024). Traditional Chinese medicine containing pyrrolizidine alkaloids or diosbulbin B are recognized as causes of DILI (Liu et al., 2024; Teschke, 2014). Wang et al. showed that glycyrrhetinic acid exerts protective effects against PA-induced liver injury in rats by potentiating the Nrf2-mediated antioxidant system through the phosphatidylinositol 3-kinase (PI3K)/protein kinase B (Akt)/glycogen synthase kinase 3 beta (GSK3β) pathway (Wang et al., 2022). In addition, glycyrrhetinic acid can inhibit the metabolic activation of diosbulbin B, thereby reducing its hepatotoxic effects in mice (Lin et al., 2023). Additionally, pretreatment with glycyrrhetinic acid significantly downregulated the expression of CYP2e1 and the high mobility group box 1(HMGB1)-toll like receptor 4 (TLR4) in APAP-exposed mice (Yang et al., 2017). Collectively, these studies suggested that glycyrrhetinic acid may have great potential in the prevention and treatment of DILI.

#### 3.3.4 Ursolic acid

Ursolic acid is a pentacyclic triterpenoid compound that is widely distributed in a variety of plants belonging to the Oleaceae, Lamiaceae, Rosaceae, and Scrophulariaceae families (Zhang and Zhu, 2011). It shows hepatoprotective effects in several liver injury models (Zheng et al., 2024; Zhou L. F. et al., 2024). Glutathione S-transferases (GSTs), as an important phase II enzyme, play a crucial role in mediating the protective effect of ursolic acid against tetrandrine-induced hepatotoxicity. Specifically, ursolic acid was found to alleviate tetrandrine-induced oxidative stress injury by competitively binding to the GST H-site pockets, thereby blocking the interaction between tetrandrine and glutathione S-transferase Mu 1 (GSTM1) (Chu et al., 2022). Although these findings suggest a novel mechanism and a potential therapeutic target for improving tetrandrine-induced hepatotoxicity, further studies are required to validate these observations and explore their clinical relevance.

#### 3.3.5 Catalpol

Catalpol is an iridoid monosaccharide found in several plants, including Rehmanniae Radix, and it exhibits antioxidant, anti-inflammatory, and anti-apoptotic capacities. Growing evidence supports its protective effects against DILI. Zhang et al. and Fu et al. demonstrated that catalpol suppresses excessive autophagy through the PERK-ATF4-CHOP pathway and synergistically activates phase-I and phase-II detoxifying enzymes via the CAR and NRF2 pathways, ultimately attenuating triptolide-induced liver toxicity (Zhang et al., 2022; Fu et al., 2020). Furthermore, based on metabolomics analyses, catalpol was found to alleviate triptolide-induced hepatic injury in mice by regulating the SIRT1/HIF-1α signaling pathway, which contributed to the restoration of hepatic glucose metabolism disorder and oxidative stress (Nie et al., 2024). These findings highlighted the therapeutic anti-DILI potential of catalpol, particularly through mechanisms related to metabolic dysfunction and oxidative stress.

#### 3.3.6 Geniposidic acid

Geniposidic acid, a natural iridoid glycoside, is a major active constituent of Gardeniae Fructus, which has been reported to alleviate liver injury through regulating bile acid and cholesterol metabolism (Song et al., 2022). As a farnesoid X receptor (FXR)-specific agonist, geniposidic acid could influence bile acid homeostasis in multiple DILI models, such as APAP-acute DILI, *Tripterygium wilfordii*-acute DILI, and *Tripterygium wilfordii*-chronic DILI. Moreover, geniposidic acid has been shown to enhance CYP-mediated bile acid metabolism and inhibit cholesterol biosynthesis via miR-19a-3p regulation (Fan et al., 2025). This dual regulatory action contributed to the restoration of hepatic metabolic balance and underscored its therapeutic potential in the treatment of DILI.

#### 3.3.7 Other terpenoids

**FIGURE 4:**
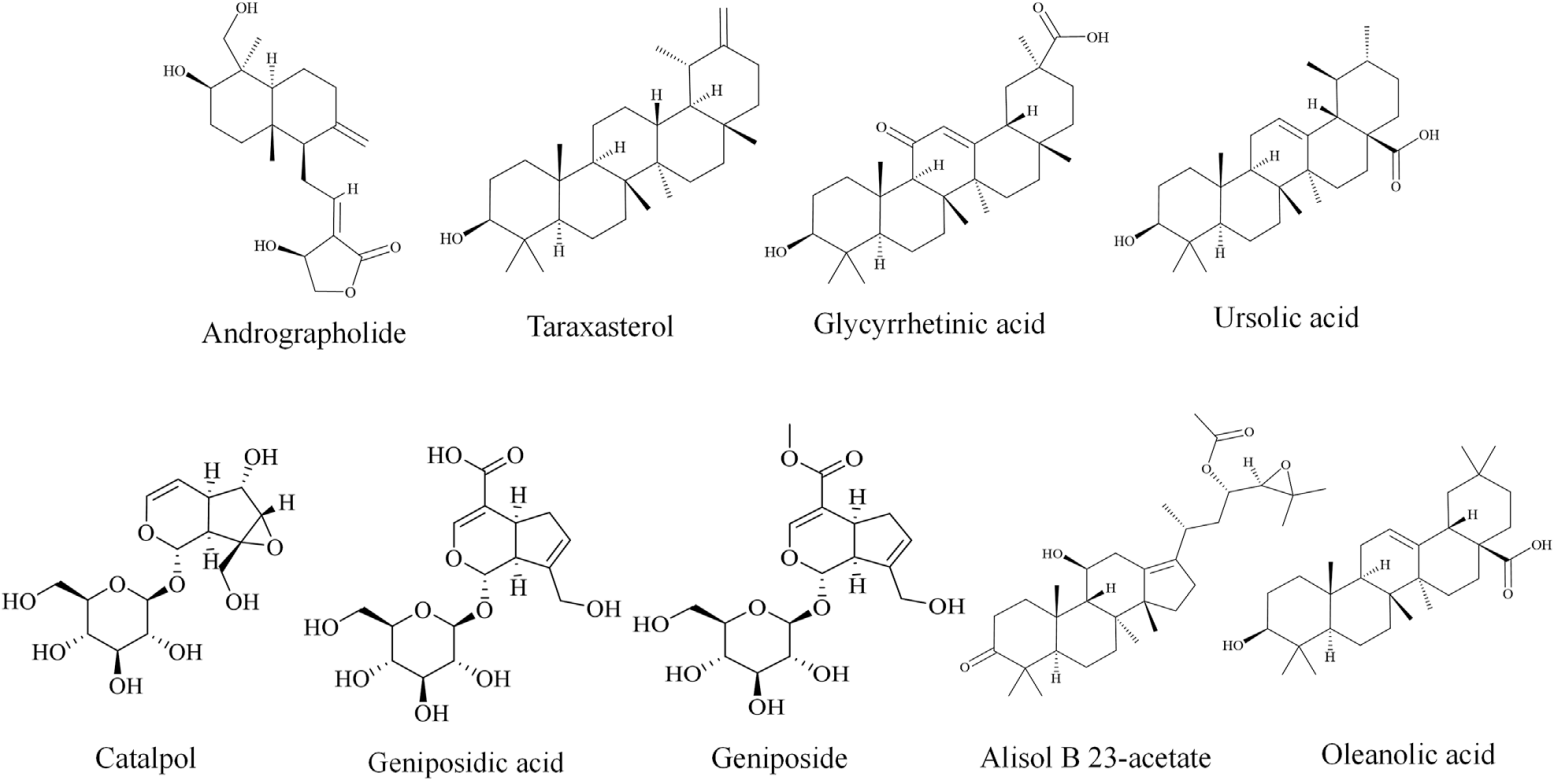
Chemical structures of terpenoids against DILI.

Other terpenoids including geniposide (Wang et al., 2016), alisol B 23-acetate (Tang et al., 2024), and oleanolic acid (Yong et al., 2019; Wang and Liu, 2024) have therapeutic potential against DILI, as listed in Table 2 and Figure 4.

### 3.4 Glycosides

Glycosides are widely distributed in plants due to their unique structural characteristics. They represent an important class of active ingredients in traditional Chinese medicine and have a wide range of biological activities, such as anti-inflammatory, antioxidant, antibacterial, anticancer, antiaging, and hepatoprotective effects (Shah et al., 2022; Alizadeh and Ebrahimzadeh, 2022; Li J. et al., 2024; Wu et al., 2020).

#### 3.4.1 Arbutin

Arbutin is a naturally occurring glucoside extracted from plants, and it exhibits various pharmacological activities. It is a bioactive polyphenol composed of a hydroquinone moiety bound to a D-glucose molecule, and it is commonly used in cosmetics and herbal dietary supplements (Wang Q. L. et al., 2024). Arbutin has shown protective effects against liver diseases (Jiang et al., 2023; Okkay et al., 2024). Administration of arbutin (25 and 50 mg/kg) for 2 weeks markedly alleviated cyclophosphamide-induced hepatotoxicity in rats. Furthermore, arbutin could suppress inflammatory markers and hepatocyte apoptosis and increase antioxidant capacity. These effects are associated with the activation of the Nrf-2/HO-1 signaling pathway (Alruhaimi, 2023). However, the underlying mechanisms through which arbutin attenuates cyclophosphamide-induced liver injury remain to be elucidated.

#### 3.4.2 Salidroside

Salidroside, a phenolic glycoside compound extracted from *Rhodiola crenulata*, used in traditional Chinese medicine, is known for its antioxidant, anti-inflammatory, anti-cancer, and anti-hypoxia effects (Zhang et al., 2021; Liang et al., 2024). Several studies have suggested that salidroside exerts protective effects in liver disease models (Hu Q. C. et al., 2021; Zhang J. Q. et al., 2020; Wu et al., 2008). Salidroside promoted sirtuin 1 (Sirt1) expression, activated the Akt/Nrf2 pathway, and inhibited the NF-kB/nucleotide-binding domain-like receptor protein 3 (NLRP3) inflammasome axis in APAP-treated L02 cells and in mice (Gao et al., 2023). Furthermore, Xu et al. found that salidroside alleviates APAP-induced liver injury via activating the AMPK/SIRT1 pathway, which is associated with the inhibition of endoplasmic reticulum stress-mediated ferroptosis in the activating transcription factor 4 (AFT4)-cation transport regulator homolog 1 (CHAC1) axis (Xu et al., 2023b). In conclusion, these findings indicated that salidroside has great potential for protecting and alleviating liver damage.

#### 3.4.3 Gastrodin

Gastrodin is the major active phenolic glycoside extracted from *Gastrodia elata* Bl., which has been widely used in the clinic. It exhibits extensive pharmacological activities such as antioxidant, anti-inflammatory, cardiovascular protective, neuroprotective, and hepatoprotective effects (Wang Y. L. et al., 2024; Xiao et al., 2023). Intraperitoneal injection of gastrodin (at concentrations ranging from 15 mg/kg to 45 mg/kg) significantly attenuated APAP-induced liver injury in mice. Gastrodin could reduce the production and release of inflammatory factors (IL-6, IL-1β, and TNF-α) and oxidative stress. The results showed that the hepatoprotective effect of gastrodin is closely related to its antioxidant and anti-inflammatory capacities, potentially involving the activation of the ERK/JUK/MAPK and Nrf2 signaling pathways (Liao et al., 2022).

#### 3.4.4 Ginsenosides

Ginsenosides, characterized by their triterpenoid glycoside structure, are the principal active components of *Panax ginseng.* To date, more than 100 types of ginsenosides have been identified and isolated from *P. ginseng* (Li Q. et al., 2024). These compounds exhibit a broad range of pharmacological activities that include, but are not limited to, anti-oxidation, anti-inflammation, immune regulation, and anticancer effects (Yu et al., 2019; Cai et al., 2022). In various pathological models of liver disease, ginsenosides have been found to show hepatoprotective effects (Yi, 2024; Li X. K. et al., 2023; Zhou H. M. et al., 2024; Guo J. A. et al., 2024). For instance, continuous treatment with ginsenoside Rg1 for 3 days enhanced the antioxidant and detoxification capacities in mice with APAP-induced liver injury, which was related to the activation of the antioxidant defense system through the Nrf2 signaling pathway (Ning et al., 2018). In addition, Bi et al. reported that ginsenoside Rg1 not only markedly decreases the levels of inflammatory cytokines TNF-α, IL-6, and IL-1β but also regulates those of apoptosis-related proteins such as Bax and Bcl-2 in APAP-treated mice. Similarly, ginsenoside Rh1 also exerted comparable hepatoprotective effects (Bi et al., 2021). Cantharidin, the active compound of Mylabris, is used as an anticancer agent. However, its clinical use is mainly limited due to hepatotoxicity (Liu et al., 2020). Research has found that ginsenoside Rb1 mitigates cantharidin-induced hepatotoxicity by inhibiting apoptosis and endoplasmic reticulum stress. Ginsenoside Rb1 could downregulate the expression of glucose-regulating protein 78 (GRP78) and inhibit the pancreatic ER kinase (PERK)-activating transcription factor 4 (ATF4), thus activating inositol-requiring enzyme 1 alpha (IRE1α) and the transcription factor 6 (ATF6) pathway (Xiong et al., 2024). Nevertheless, only a few studies have explored the protective effects of Rb1 against cantharidin-induced liver injury, and its underlying mechanisms are yet to be fully elucidated. Given their chemical diversity and potent hepatoprotective effects (Wu et al., 2025; Gao et al., 2017), ginsenosides provide a promising new insight into the development of natural products for the treatment of DILI.

#### 3.4.5 Other glycosides

**FIGURE 5:**
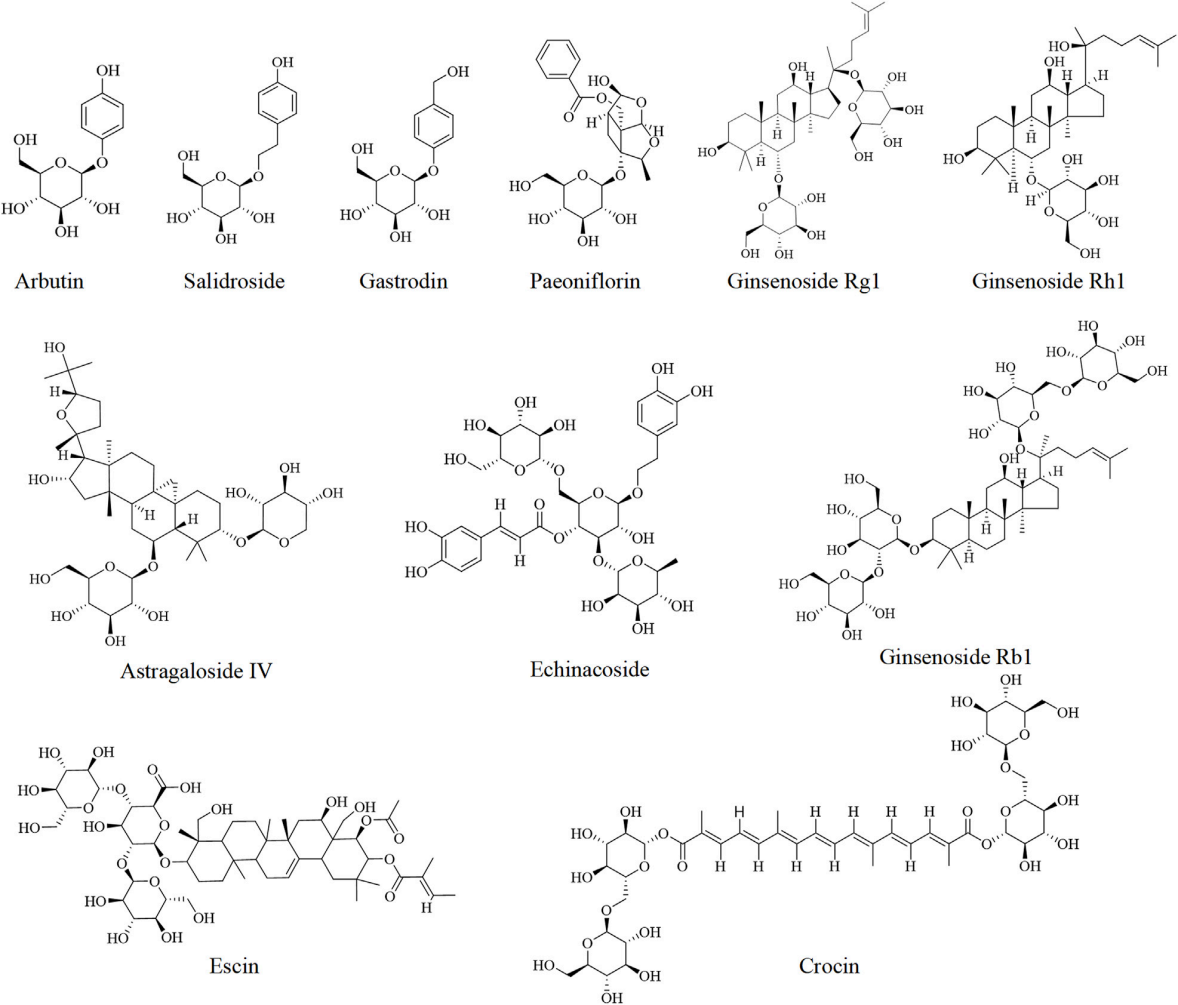
Chemical structures of glycosides against DILI.

Other glycosides including echinacoside (Thida et al., 2021), paeoniflorin (Deng et al., 2024; 2022), notoginsenosides (Tian et al., 2024), escin (Lee et al., 2019), crocin (Sokar et al., 2022), and astragaloside IV (Guo Y. T. et al., 2024) have shown effectiveness in the prevention and treatment of DILI, which are listed in Table 2 and Figure 5.

## 4 Discussion and perspectives

DILI is an infrequent but serious adverse reaction to drugs or other xenobiotics. It may occur either as a predictable event when an individual is exposed to toxic doses of some drugs or as an unpredictable event with many commonly used medications (Andrade et al., 2019). DILI is a significant issue that needs to be taken seriously during the development and application of drugs. Although the concept of DILI was proposed several decades ago (Allison et al., 2023), more efforts are still needed for its investigation. Natural products, as the primary ingredients of traditional Chinese medicine, have become valuable resources for the development of novel pharmaceuticals due to their low toxicity and side effects, extensive and strong bioactivities, and abundant availability.

**FIGURE 6:**
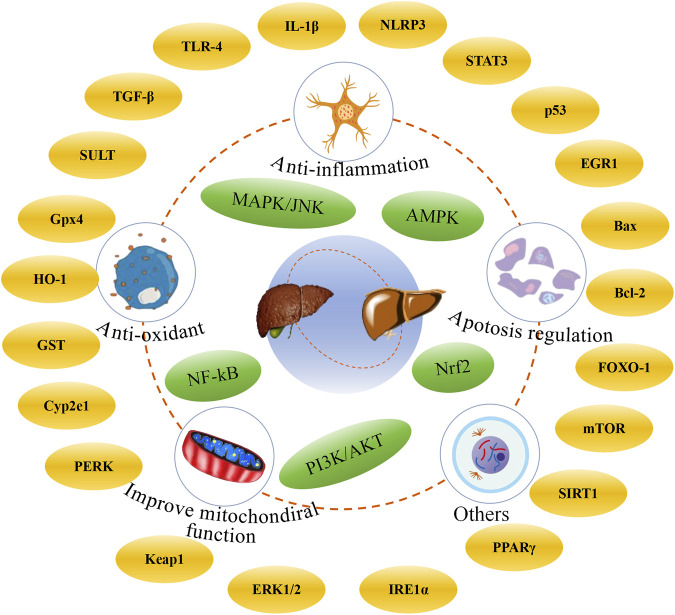
Potential integrated mechanisms of natural products against DILI.

In this review, we systematically summarized the recent advances in natural products with potential anti-DILI effects (including flavonoids, phenylpropanoids, terpenoids, and glycosides) over the past 5 years, and the mechanisms of hepatoprotective effects were also discussed. It is widely recognized that these compounds exhibit significant antioxidant and anti-inflammatory properties, which alleviate the symptoms of DILI, such as cellular inflammation and cholestasis, which frequently occur in the clinic (Björnsson and Björnsson, 2022). The mechanisms involve multiple pathways and targets, such as anti-oxidation, anti-inflammation, improvement of mitochondrial function, and inhibition of apoptosis (Figure 6). However, the application of natural products in the prevention and treatment of DILI is associated with several notable limitations and challenges. First, it should be noted that many studies have only been conducted in preclinical animal models, and the clinical evidence remains limited. Moreover, the intricate signaling pathways involved in natural products for the prevention and treatment of DILI require further exploration to fully understand the mechanism of action of natural products. Second, compared with other liver diseases, researchers have conducted relatively fewer studies on DILI in recent years. The emergence of such phenomena might be related to public awareness, supervision of market, and policies. DILI is a complex condition and is influenced by multiple risk factors, especially idiosyncratic DILI. These factors include, but are not limited to, age, gender, genetic, environment, and disease state (Allison et al., 2023). As the liver is the primary site of medications metabolism, DILI still represents an inescapable and potentially fatal challenge in the evaluation of drug safety. Although APAP cannot exclusively cause liver damage, we found that APAP-induced liver injury with reproducibility, stability, and clinical relevance is one of the most common models to evaluate the potential of natural products against DILI (Jaeschke and Ramachandran, 2024; Stravitz and Lee, 2019; Jaeschke et al., 2013). Nevertheless, it is noteworthy that hepatotoxic drugs, including anti-cancer drugs such as doxorubicin, tamoxifen, and methotrexate, and traditional Chinese medicines containing pyrrolizidine alkaloids, tetrandrine, and diosbulbin B, are attracting increasing attention in the fields of toxicology, public health, nutrition, and food science. In addition, it is crucial to focus not only on the intrinsic effects of the drugs themselves but also on their potential interactions.

Therefore, based on the above discussion, we should (1) increase the screening of bioactive compounds and the investigation of their mechanisms against DILI; (2) establish stable and mature animal models for a variety of DILIs and investigate the individual differences in DILIs by diverse genetic models to explore the genetic variability; (3) enhance collaboration between clinical application and fundamental research to bridge the gap between them; (4) study the biomarkers of DILI patients for future research on natural products-based hepatoprotection.

## 5 Conclusion

In summary, while natural products have considerable potential in the prevention and treatment of DILI, overcoming the limitations and challenges still requires sustained research efforts and collaborative endeavors. With continued in-depth research, it is possible to develop more natural products and their derivatives that are effective, efficient, cheaper, and have low side-effects for the prevention and treatment of DILI and other diseases.
